# Orchestrating neuronal networks: sustained after-effects of transcranial alternating current stimulation depend upon brain states

**DOI:** 10.3389/fnhum.2013.00161

**Published:** 2013-04-30

**Authors:** Toralf Neuling, Stefan Rach, Christoph S. Herrmann

**Affiliations:** ^1^Experimental Psychology Lab, University of OldenburgOldenburg, Germany; ^2^Research Center Neurosensory Science, University of OldenburgOldenburg, Germany

**Keywords:** EEG, transcranial alternating current stimulation, tACS, alpha, brain state

## Abstract

The interest in transcranial alternating current stimulation (tACS) has significantly increased in the past decade. It has potential to modulate brain oscillations in a frequency specific manner, offering the possibility to demonstrate a causal nature of oscillation behavior relationships. TACS is a strong candidate as a tool for clinical applications, however, to fulfill this potential, certain parameters have yet to be evaluated. First, little is known about long-lasting after-effects of tACS with respect to the modulations of rhythmic brain activity. Second, the power of endogenous brain oscillations might play a crucial role in the efficacy of tACS. We hypothesize that the after-effects of tACS depend on the endogenous power of oscillations. To this end, we modulated the power of endogenous occipital alpha oscillations via tACS. In two experiments, participants either had their eyes open or closed to keep endogenous alpha power either low or high while they were stimulated for 20 min with their individual alpha frequency (IAF) and simultaneously performing a vigilance task. After-effects on IAF power were evaluated over a course of 30 min with a pre stimulation period serving as baseline. After-effects were strongly dependent on IAF power. Enhanced IAF power was observed for at least 30 min after tACS under conditions of low endogenous IAF power, whereas, IAF power could not be further enhanced by tACS under conditions of high IAF power. The current study demonstrates, for the first time, a long lasting effect after tACS on endogenous EEG power in the range of the stimulation frequency. Additionally, we present conclusive evidence that the power of the endogenous oscillations has a critical impact on tACS efficacy. Long lasting after-effects foster the role of tACS as a tool for non-invasive brain stimulation and demonstrate the potential for therapeutic application to reestablish the balance of altered brain oscillations.

## Introduction

Brain oscillations play a crucial role in motor, perceptual, and cognitive processes (Başar et al., 2001; Herrmann et al., 2004; Buzsáki, 2006; Schroeder and Lakatos, 2009) and alterations of these oscillations can be linked to psychiatric disorders (Herrmann and Demiralp, 2005; Uhlhaas et al., 2008). Previous experiments demonstrated associations by correlating behavior and brain oscillations. However, non-invasive brain stimulation techniques, combined with electroencephalography (EEG), offer the possibility to demonstrate a *causal* relationship (for recent reviews see: Thut et al., 2011a; Miniussi et al., 2012).

Oscillatory brain activity is evoked by neuronal network activity of different spatial scales (Buzsáki and Draguhn, 2004). These oscillations, which manifest as changes of extracellular electric fields, in turn, can serve as a feedback signal to structure the activity of the neurons that generated it (Fröhlich and McCormick, 2010). Transcranial electrical stimulation (TES) with weak sinusoidally varying currents is a non-invasive brain stimulation technique that can mimick endogenous electric fields and is thought to directly modulate ongoing oscillatory brain activity as suggested by numerous studies (e.g., Marshall et al., 2006; Antal et al., 2008; Kanai et al., 2008; Pogosyan et al., 2009; Zaehle et al., 2010; Feurra et al., 2011; Neuling et al., 2012a; Polania et al., 2012). Oscillating TES includes transcranial alternating current stimulation (tACS) and tACS with a DC-offset as referred to as oscillating transcranial direct current stimulation (otDCS), see Herrmann., et al. (in revision, this issue) for an overview. TACS/otDCS produces periodic changes of cortical excitability over time with a specific frequency. As a consequence, the power of the spontaneous brain activity in the range of the stimulation frequency can be enhanced (Marshall et al., 2006; Zaehle et al., 2010; Neuling et al., 2012a).

Although immediate after-effects on brain activity have been demonstrated via EEG, the duration and contributing parameters of these after-effects remain largely unknown. This knowledge would provide not only a foundation for further neuroscientific studies on the causal relevance of brain oscillations, but also for clinical applications to re-establish a balance in altered brain oscillations (Kuo and Nitsche, 2012). Only by determining the long term after-effects of tACS, it is possible to work toward a successful treatment of dysfunctional brain oscillations.

It has been argued that only physiologically meaningful brain rhythms can be entrained (Thut et al., 2011a). Based on this view, brain rhythms can either be entrained depending on the *state* (ongoing) or *function* (task-related) of the oscillations. With regard to state, this means, for example, that sleep-like slow wave oscillations can be entrained in sleep (Marshall et al., 2006) but not in waking state (Bergmann et al., 2009) and stimulation in the alpha rhythm is more effective in the dark compared to beta stimulation, whereas it is opposite in light (Kanai et al., 2008). Entrainment of functional rhythms includes the modulation of task-related oscillations (Romei et al., 2010; Thut et al., 2011b; Brignani et al., 2013). Although the current study focuses on the state-dependency of physiological tACS after-effects, the results are expected to generalize to task-related oscillations, as we assess general properties of oscillations.

In the context of synchronization the problem is: “Under which conditions the observed […] frequency of oscillations in an externally periodically driven system will come into coincidence with the frequency of the driving? Usually these conditions are quantified in terms of the power and the mismatch between the frequency of the external force and the natural (internal) frequency of the oscillator” (Osipov et al., 2007, pg. 35). This means that a neuronal network with strong oscillatory power should not be as prone to a certain external periodic force as a network with weaker oscillatory power. *In vitro* experiments revealed that neuronal network activity can be entrained by sinusoidal electric fields with an intensity similar to that of the endogenous electric field of that network (Fröhlich and McCormick, 2010). The same study also found that entrainment works best if the external and internal frequency match. It would be desirable to demonstrate this interaction of the external and internal oscillation with tACS to contribute to the understanding and feasibility of this brain stimulation method.

The goal of this study was to discern for how long endogenous brain oscillations are enhanced post tACS and how the baseline power of the endogenous oscillations modulates this effect. To this end, we conducted two experiments focusing on the occipito-parietal alpha rhythm (8–12 Hz) that was recorded via EEG. Participants either had their eyes closed or open (in a dark room) while they received tACS with their individual alpha frequency (IAF). Without any visual stimulation (closed eyes), the endogenous alpha power is increased compared to visual stimulation (open eyes) as already discovered by Berger (1929). We hypothesize that this experimental modulation of endogenous alpha power results in a weak after-effect of tACS on endogenous alpha power when participants keep their eyes closed and a comparatively strong after-effect if participants keep their eyes open.

## Methods

Two experiments were conducted in which participants had their eyes either closed (Experiment 1) or open (Experiment 2). Except for participants, all materials and methods were the same for both experiments.

### Participants

Subjects gave written informed consent before participation. Participants were university students and were paid for participation. All participants were medication-free at the time of the experiments. They reported no hearing deficits, presence or history of epilepsy, neurological or psychiatric disorders, cognitive impairments, intracranial metal or cochlear implants. All participants were right handed, according to the Edinburgh handedness inventory (Oldfield, 1971). In a single blind study design, participants were assigned to either experimental (*stim*) or control group (*sham*). In a debriefing after the experiment, participants were informed about the hypotheses and whether they belonged to the *stim* or *sham* group. The experimental protocol was approved by the ethics committee of the University of Oldenburg and was conducted in accordance with the Declaration of Helsinki.

#### Experiment 1: eyes closed

Twenty-four healthy, right-handed subjects participated in the study. Four participants were excluded due to technical problems which corrupted the EEG data. One participant was excluded because the sensation threshold of the stimulation was as low as 0.1 mA. Nineteen subjects (12 female) with an age of 22.9 ± 0.8 (mean ± standard error of the mean, SEM) years were used for data analysis. *Stim* and *sham* groups did not differ significantly in age (stim: 23.5 ± 1.4 years; sham: 22.3 ± 1.1 years; independent *t*-test: *t*_17_ = 0.68, *P* = 0.51), gender (stim: 7 female; sham: 5 female, χ^2^_1_ = 0.43 (*n* = 19), *P* = 0.52), individual alpha frequency (IAF) (stim: 9.9 ± 0.3 Hz; sham: 10.3 ± 0.3 Hz, independent *t*-test: *t*_17_ = 1.01, *P* = 0.33), or threshold (see below) (stim: 905 ± 122 μA; sham: 844 ± 35 μA, independent *t*-test: *t*_17_ = 0.58, *P* = 0.57).

#### Experiment 2: eyes open

Thirty healthy, right-handed subjects participated in the study. One participant reported in the post experiment interview that he kept his eyes closed for most of the time. To take account of other participants who might not have reported that they left their eyes closed, we z-transformed the participant's mean IAF power in the post stimulation period. A high z-score indicates that this value deviates from the distribution of the other values. All participants with a z-score higher than 1.65 (α < 0.05, one-tailed) were rejected from further analyses. Twenty-two subjects (12 female) with a mean age of 25.1 ± 0.6 years remained for data analysis. *Stim* and *sham* group did not significantly differ in age (stim: 24.2 ± 0.6 years; sham: 26.1 ± 1.1 years; independent *t*-test: *t*_20_ = 1.53, *P* = 0.14), gender (stim: 5 female; sham: 7 female, χ^2^_1_ = 0.73 (*n* = 22), *P* = 0.39), IAF (stim: 10.3 ± 0.2 Hz; sham: 10.3 ± 0.5 Hz, independent *t*-test: *t*_20_ = 0, *P* = 1.00), and threshold (see below) (stim: 877 ± 48 μA; sham: 1200 ± 154 μA, independent *t*-test: *t*_20_ = 1.996, *P* = 0.06).

### EEG

The experiments were performed in a dark room with the participants seated in a recliner. The EEG was measured from 25 sintered Ag-AgCl electrodes mounted in an elastic cap (Easycap, Falk Minow, Munich, Germany) with a standard 10–20 system layout, and vertical EOG, referenced to the nose. The ground electrode was positioned on the forehead at Fpz. Electrode impedance was kept below 10 kΩ. Signals were recorded using Brain Vision Recorder (Brain Products GmbH, Gilching, Germany) with an online low pass filter (250 Hz). When an electrode reached 70% saturation, a DC reset was applied. Sampling rate was 500 Hz and amplified in the range of ±3.2768 mV at a resolution of 0.1 μV. Stimulus markers and EEG data were digitally stored on hard disk for further offline analysis. No offline filters were applied.

### Electrical stimulation

**Figure 1:**
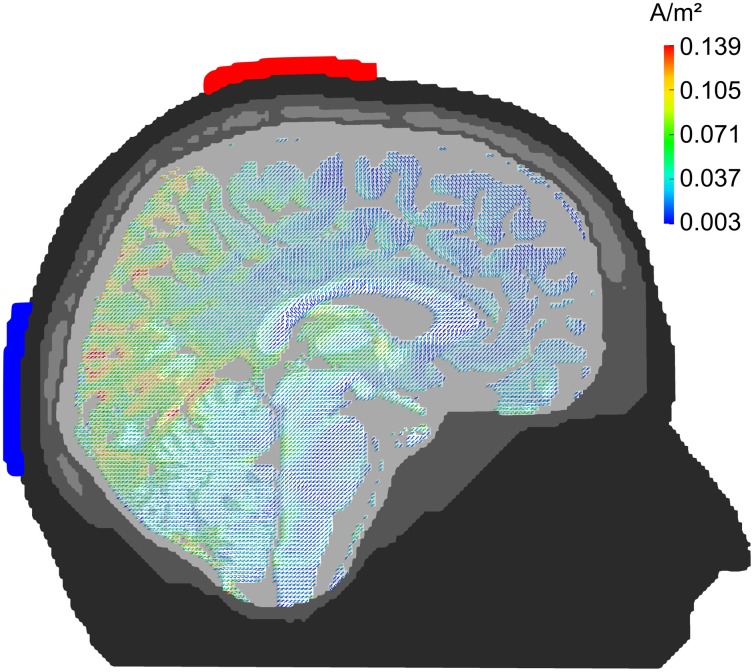
**Current density distribution.** Results of a finite-element model simulation of tACS (Neuling et al., 2012a). Simulation electrodes are centered at electrode positions Cz and Oz of the 10/20 system. Current densities are highest in the posterior cortex.

**Figure 2:**
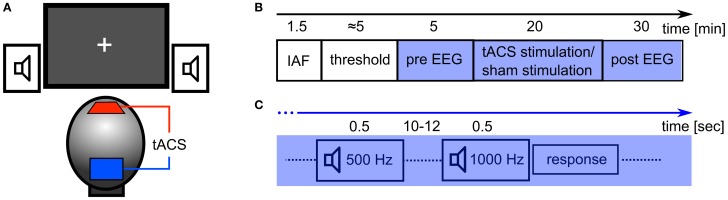
**Experimental details. (A)** Experimental setup: tACS electrodes were centered at electrode positions Cz and Oz of the 10/20 system. Subjects were seated in front of a computer screen with a gray fixation cross on a black background (Experiment 2). Auditory stimuli were delivered through stereo speakers. **(B)** Sequence of the experiment: at first, the participant's IAF was obtained with their eyes closed, before the thresholds for skin sensation and phosphene perception were acquired. In the following 55 min, participants had to perform a detection task with either closed (Experiment 1) or open eyes (Experiment 2). In the first 5 min of this period, the EEG was recorded (*pre* EEG) and served as a baseline. Subsequently, 20 min of stimulation (tACS or sham) were applied. Afterwards, the EEG was recorded for 30 min (*post* EEG). **(C)** Detection task: every 10–12 s, a tone was presented for 500 ms. 80% of those were standards (500 Hz) and 20% were targets (1000 Hz). Subjects had to respond with a button press to each presentation of the target stimulus.

The tACS was applied via two surface conductive-rubber electrodes (5 × 7 cm) enclosed in saline-soaked sponges (Neuroconn, Ilmenau, Germany) centered at Cz and Oz underneath the EEG recording cap (see Figure 2A). Stimulation electrode positions were chosen in order to affect the occipital cortex (Figure 1). The impedance was kept below 10 kΩ. An alternating, sinusoidal current at the IAF of each participant was applied using a battery-operated stimulator system (Eldith, Neuroconn, Ilmenau, Germany). The intensity of the sinusoidal current was adjusted individually to the highest intensity at which the stimulation was not noticed by the participants. To obtain this threshold, we started with an intensity level of 1500 μA (peak-to-peak). If the subject indicated no skin sensation or phosphene perception, we increased the intensity in steps of 100 μA. As soon as the participant either indicated skin sensation or phosphene perception, we decreased the intensity in steps of 100 μA. Each intensity step was applied for approximately 20 s, without fade-in/out. The obtained threshold level was used as stimulation intensity. The experimental group received 20 min of stimulation. In the beginning, and at the end, the stimulation was faded-in and faded-out for 10 s. In the control group, sham stimulation was applied. While all other stimulation parameters were the same as in the experimental group, the control group received only 30 s of stimulation, a procedure that has been used in previous studies (e.g., Polania et al., 2012).

### Design

The experimental procedure is illustrated in Figure 2B. After the EEG and TES electrodes were attached, the participant's IAF was estimated. Participants were asked to keep their eyes closed while the spontaneous EEG was recorded for 90 s. Afterwards, raw EEG data of electrode Pz was split into one-second segments. Segments containing artefacts were rejected. A fast Fourier transformation (FFT) was performed on the first 50 artifact-free segments and the resulting spectra were averaged. The power peak in the alpha range (8–12 Hz) was considered as IAF and used as stimulation frequency. Subsequently, the stimulation intensity was determined. Now, the EEG was recorded for 5 min which served as a baseline (*pre*-EEG), before tACS or sham stimulation was applied for 20 min. Afterwards, the EEG was recorded for 30 min (*post*-EEG).

During EEG recording, the subjects performed a simple auditory detection task and were instructed to either close their eyes (Experiment 1) or leave their eyes open (Experiment 2). In Experiment 2, participants were asked to fixate on a cross that was presented in the center of a computer screen. The auditory detection task was introduced to ensure vigilance during the course of the experiment (Figure 2C). With an inter stimulus interval of 10 to 12 s a tone was presented for 500 ms via loudspeakers. In 80% of the presentations this was a 500 Hz tone and in 20% of the presentations this was a 1000 Hz tone. Subjects were instructed to press a button when the 1000 Hz tone was presented.

After the experiment, participants were at first asked to guess if they were stimulated at all or perceived phosphenes during the stimulation and were subsequently debriefed. Additionally, a questionnaire assessed possible adverse effects.

### Questionnaire on adverse effects

To obtain possible adverse effects for tACS a translated version of a questionnaire introduced by Brunoni et al. (2011) was used. The following side-effects were inquired: headache, neck pain, scalp pain, tingling, itching, burning sensation, skin redness, sleepiness, trouble concentrating and acute mood change. Specifically, participants were asked to indicate the intensity of the side-effect (1, absent; 2, mild; 3, moderate; 4, severe) and if they attributed the side-effect to the tACS (1, none; 2, remote; 3, possible; 4, probable; 5, definite).

### Data analysis

Data analysis was performed using MATLAB R2012a (The MathWorks Inc, Natick, MA, USA) and EEGLAB 11.0.4.3 (Delorme and Makeig, 2004). For statistical analysis, SPSS 20.0 (IBM Corp, Armonk, NY, USA) was used.

#### EEG data

Data analysis was conducted in accordance with the approach introduced by Zaehle et al. (2010). The *post*-EEG was divided into 10 epochs of 3 min each. Each of the data epochs (*pre*-EEG, 10 × *post*-EEG) from each participant was split into 180 one-second segments. Segments containing artefacts (eye movements, muscle activity), acoustical stimulation or a motor response were rejected from further analysis. The first 120 segments of each data set without artefacts were used for further analysis. After subtracting the mean value of each segment to avoid DC distortion of the spectra at 0 Hz, an FFT was applied on each segment and the resulting 120 spectra for each data set were averaged. The parieto-occipital electrode Pz was chosen for power analysis. To evaluate power changes in the range of the IAF ± 2 Hz, the individual mean spectral powers were calculated. In order to account for individual differences, the power data were normalized to the alpha-power of the pre-EEG measurement. To discern the specificity of the power changes, we additionally analyzed frequency bands below (*lower band*, IAF −5 to −3 Hz) and above (*upper band*, IAF +3 to +5 Hz) the IAF.

A recent study conducted by our group aimed to reveal differences in coherence before and after tACS (Strüber et al., under revision). Therefore, we calculated changes of coherence in the alpha-range (7.8–11.7 Hz). We computed the mean magnitude squared coherence (Equation 1) between EEG electrodes P3 and P4 in the *pre*-EEG, which served as a baseline.

$$C_{xy}(f)=\frac{|P_{xy}(f){|}^{2}}{P_{xx}(f)P_{yy}(f)}$$

The magnitude squared coherence (C) for a specific frequency (f) is a function of the power spectral densities of two signals x (P3) and y (P4) and the cross power spectral density of the two signals (P_*xy*_(f)). The coherence ranges between 0 (no coherence) and 1 (perfect coherence). In order to account for individual differences, the average coherence for each *post*-EEG epoch was normalized to the individual coherence baseline.

For statistical analysis of the tACS after-effect, normalized spectral power/ coherence was entered into a Two-Way analysis of variance (ANOVA) with repeated measurements with between subject factor *group* (2 levels) and within subject factor *time* (10 levels). In case Mauchly's test detected violation of sphericity, Greenhouse-Geisser corrected values are reported. This applies for all ANOVAs conducted. To further elaborate on the duration of the power after-effect, we examined when the alpha-power returned to baseline. Therefore, one sample *t*-tests against 1, which represents the baseline alpha power, were conducted. This means, if the mean of the relative alpha-power is 1 after stimulation, the power does not differ from the baseline power and the *t*-test against 1 is not significant. Bonferroni correction was applied to account for multiple comparisons.

To discern whether or not alpha topographies after tACS differed between tACS and sham groups, we calculated Spearman's rank correlation coefficients between the relative IAF power between tACS and sham groups for each electrode, whereby a high correlation would indicate a similar topography. To assess, whether or not an increase in IAF power from pre to post stimulation was locally specific, we chose a subset of electrodes (frontal: Fz, temporal: FT9/FT10, parietal: Pz) based on their position relative to the stimulation electrodes and entered the normalized IAF power into a Two-Way ANOVA with repeated measurements with between subject factor *group* (2 levels) and within subject factor *electrode* (4 levels). For *post-hoc* analyses, Bonferroni corrected 2-sample *t*-tests (one-tailed) were applied.

#### Behavioral data

We analyzed the behavioral performance of the auditory detection task for the *pre*-block, the *stimulation*-block and the *post*-block. Reaction times exceeding 1500 ms were considered as misses and excluded from further analysis. Furthermore, we calculated the sensitivity index *d'* (Wickens, 2001), the difference between the *z*-transformed hit rate and the false alarms rate. In the stimulation and post block, the same number of targets and standards as presented in the pre block were randomly chosen. We entered the behavioral data into Two-Way ANOVAs with repeated measurements with between subject factor *group* (2 levels) and within subject factor *block* (3 levels).

### Questionnaire

To compare *stim* and *sham* group, individual responses for each item were entered into a Mann–Whitney *U* test.

## Results

### Experiment 1: eyes closed

#### Debriefing

None of the participants indicated phosphenes during stimulation. Twenty-six percent of the participants indicated that they assumed to be stimulated. The judgments did not differ between the *stim* and the *sham* group (stim: 20%, sham: 33%, χ^2^_1_ = 0.15 (*n* = 19), *P* = 0.70). None of the responses on the items of the questionnaire differed between *stim* and *sham* group (Mann–Whitney *U* test: for all responses, *p* > 0.05).

Most common symptoms among the participants were *Sleepiness* (89%) and *Concentration* (63%). Other common symptoms were *Headache* (26%), *Neck pain* (26%), *Scalp pain* (37%), *Tingling* (26%), and *Itching* (16%). Single subjects indicated *Burning sensation*, *Skin redness*, and *Acute mood change*. Qualitative analysis of the responses revealed that participants did not attribute the adverse effects to the tACS, but to the experimental setting (*dark room; Headache, Sleepiness, Concentration*), design (“monotonous task”: *Sleepiness, Concentration*) and the fluids below the electrodes (“EEG gel” and “saline solution”; *Tingling, Itching, Burning sensation, Skin redness*).

#### Behavioral data

**Figure 3:**
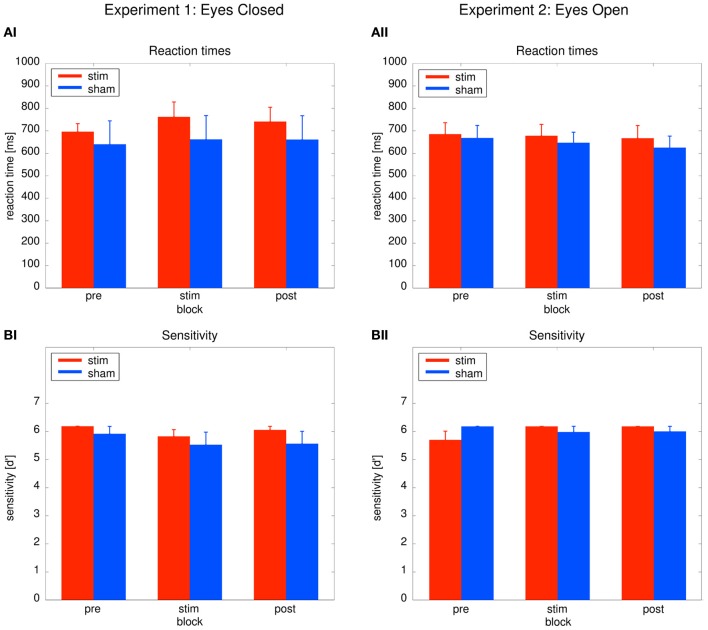
**Behavioral results.** Left: Experiment 1, right: Experiment 2; red: tACS stimulation group, blue: sham stimulation group. **(AI,AII)** Reaction times: average reaction times for the *stim* and the *sham* group for the three blocks of the detection task. Bars indicate the mean ± SEM. **(BI,BII)** Sensitivity *d'*: average *d'* for the *stim* and the *sham* group for the three blocks of the detection task. Bars indicate the mean ± SEM.

As depicted in Figure 3AI, reaction times did not differ between groups or across blocks. Behavioral effects were tested with a Two-Way ANOVA with repeated measurements with the between subject factor *group* (2 levels) and the within subject factor *time* (3 levels: *pre, stim, post*). The ANOVA revealed no significant effects (*group*: *F*_1_ = 0.31, *P* = 0.59; *time*: *F*_1.39_ = 0.12, *P* = 0.81; *group × time*: *F*_1.39_ = 1.104, *P* = 0.33). Sensitivity analysis demonstrated that both groups were able to perform the detection task. Sensitivity was not significantly different between both groups (Figure 3BI). An ANOVA yielded no significant results (*group*: *F*_1_ = 0.99, *P* = 0.33; *time*: *F*_2_ = 2.17, *P* = 0.13; *time* × *group*: *F*_2_ = 0.23, *P* = 0.799). The absence of significant behavioral differences between the groups confirm that sham stimulation was successful.

#### Electrophysiological data

**Figure 4:**
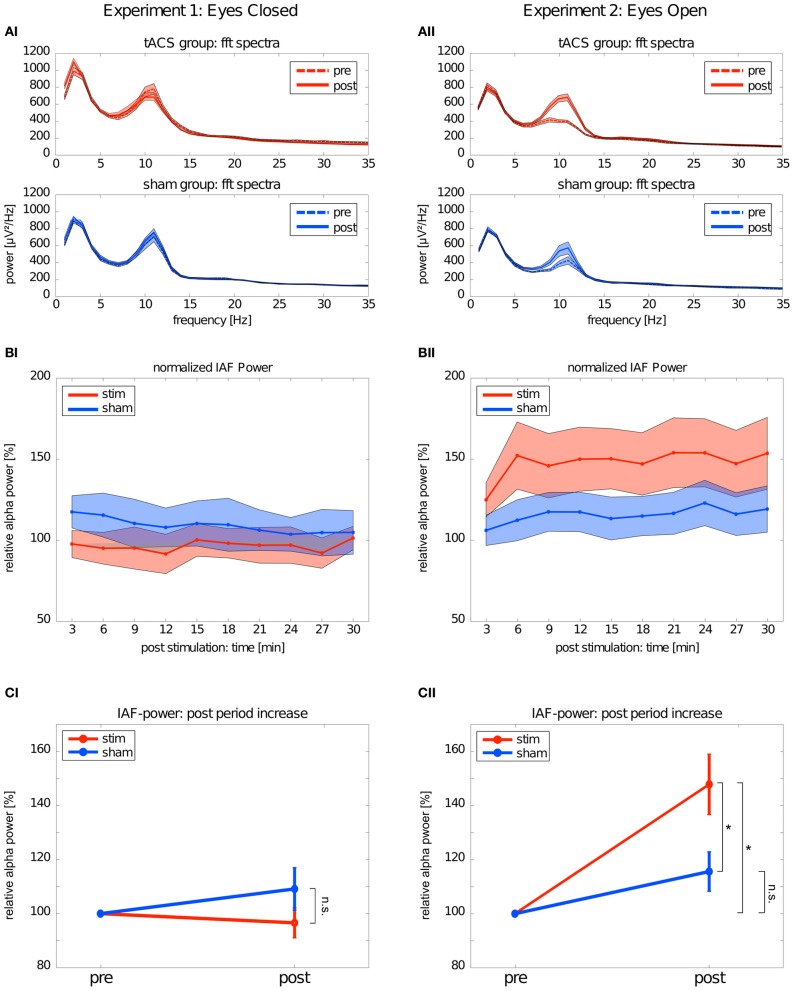
**Changes in power.** Left: Experiment 1, right: Experiment 2; red: tACS stimulation group, blue: sham stimulation group. **(AI,AII)** Average power spectra: average FFT power spectra for the *pre* EEG and the *post* EEG. Solid lines depict the mean, shaded areas depict the SEM. **(BI,BII)** Timecourse of IAF power after stimulation: change of the IAF power over the whole *post* EEG block. Solid lines depict the mean, shaded areas depict the standard deviation. **(CI,CII)** Average IAF power before and after stimulation: change of the average normalized IAF power from *pre* EEG to *post* EEG (mean ± SEM). Asterisks depict significant differences.

**Figure 5:**
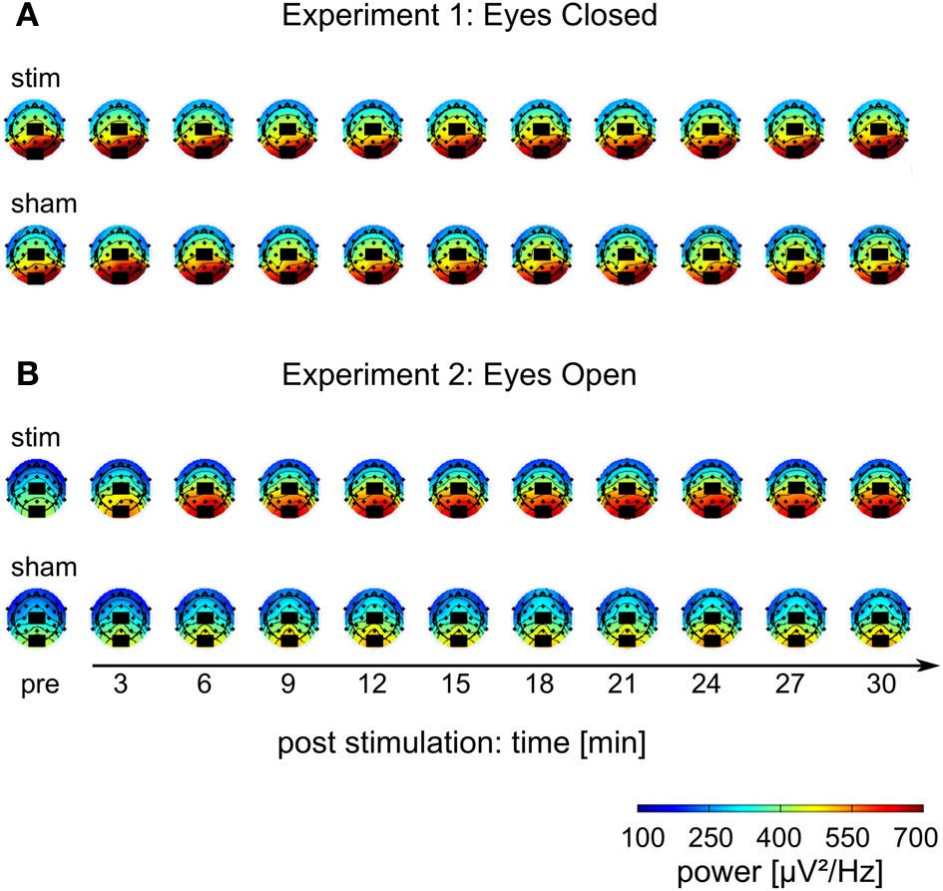
**Topographies of IAF power. (A)** Experiment 1, **(B)** Experiment 2; colors depict power. Black rectangles illustrate the placement of the stimulation electrodes.

The mean FFT power spectra of the *pre* and *post* EEG reveal no difference between *stim* and *sham* group in any frequency band (Figure 4AI). The timecourse of the IAF power (Figure 4BI) and the corresponding topographies (Figure 5A) do not differ between groups (*r* = 0.98, *P* < 0.001). A Two-Way ANOVA with repeated measurements on the normalized IAF power with the between subject factor *group* (2 levels) and the within subject factor post EEG *time* (10 levels) was conducted. The ANOVA revealed no significant main effects or interactions (*group*: *F*_1_ = 1.75, *P* = 0.20; *time*: *F*_2.58_ = 1.09, *P* = 0.36; *group × time*: *F*_2.58_ = 1.01, *P* = 0.39). Likewise, no *group* effects were found for the *lower* (*F*_1_ = 2.273, *P* = 0.15) or *upper* frequency band (*F*_1_ = 0.002, *P* = 0.97).

Subsequently, one sample *t*-tests against 1 (corresponding to baseline IAF power) were performed to test for a power increase after tACS and sham stimulation (Figure 4CI) compared to baseline IAF power, which revealed no significant power increase after tACS (*stim*: *t*_9_ = 0.61, *P* = 0.55) or sham stimulation (*sham*: *t*_8_ = 0.16, *P* = 0.28). Similar results were found for the *lower* (*stim*: *t*_9_ = 0.89, *P* = 0.40; *sham*: *t*_8_ = 1.10, *P* = 0.30) and *upper* frequency band (*stim*: *t*_9_ = 1.72, *P* = 0.12; *sham*: *t*_8_ = 0.55, *P* = 0.59).

**Figure 6:**
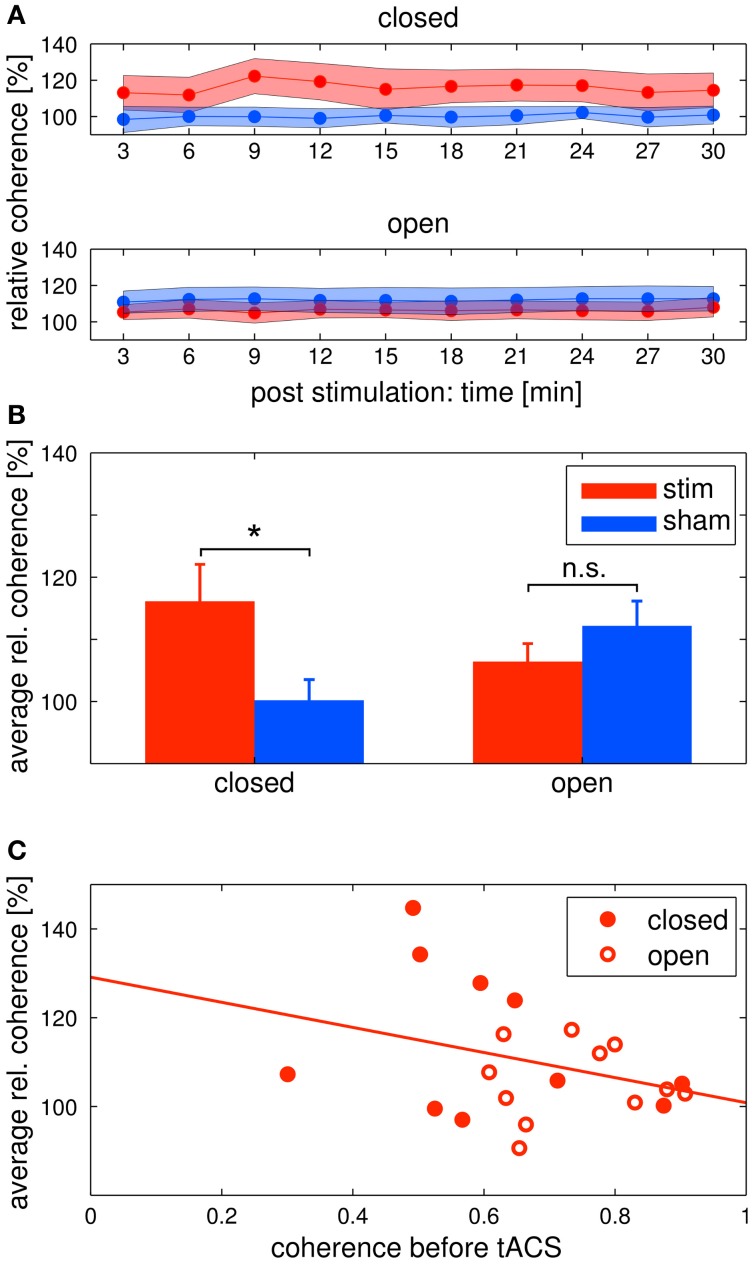
**Changes in alpha coherence.** Red: tACS stimulation group, blue: sham stimulation group. **(A)** Timecourse of coherence after stimulation: change of the alpha coherence over the whole *post* EEG block. Solid lines depict the mean, shaded areas depict the standard deviation. Upper: closed eyes, lower: open eyes. **(B)** Relative difference in coherence after stimulation: average alpha coherence of the whole *post* EEG block. Solid lines depict the mean, shaded areas depict the standard deviation. Bars indicate the mean ± SEM, asterisks indicate significant differences. In the closed eyes condition, alpha coherence was increased in the tACS stimulation group compared to the sham stimulation group. **(C)** Correlation of *pre* alpha coherence and coherence chance *post* stimulation: lower pre stimulation coherence yields higher coherence increases after tACS. Red discs: participants of the eyes closed experiment, red circles: participants of the eyes open experiment. The solid red line depicts the regression curve.

Alpha coherence was increased in the *stim* group compared to the *sham* group (Figures 6A upper/B left). This was confirmed by a Two-Way ANOVA with repeated measurements on the normalized alpha coherence with the between subject factor *group* (2 levels) and the within subject factor post EEG *time* (10 levels). The ANOVA revealed a significant main effect of *group* (*F*_1_ = 5.41, *P* = 0.03, η^2^ = 0.24). Neither a main effect of *time* (*F*_3.63_ = 1.76, *P* = 0.15) nor a significant interaction *group × time* (*F*_3.63_ = 1.59, *P* = 0.19) were observed.

### Experiment 2: eyes open

#### Debriefing

As in the eyes closed condition, no participant indicated phosphene perception during stimulation. Overall, 50% of the participants indicated that they assumed to be stimulated. The responses did not differ between the *stim* and the *sham* group (stim: 45%, sham: 54%, χ^2^_1_ = 0.67 (*n* = 22), *P* = 0.67). None of the responses on the items of the questionnaire differed between *stim* and *sham* group (Mann–Whitney *U* test: for all responses, *p* > 0.05).

Most common symptoms among the participants were *Sleepiness* (45%) and *Concentration* (36%). Single subjects indicated *Headache, Tingling, Burning sensation* and *Skin redness*. Qualitative analysis of the responses revealed that participants did not attribute the adverse effects to the tACS, but to the experimental setting (*dark room; Headache, Sleepiness, Concentration*), design (“monotonous task”: *Sleepiness, Concentration*) and the fluids below the electrodes (“EEG gel” and “saline solution”; *Tingling, Itching, Burning sensation, Skin redness*).

#### Behavioral data

Performance between the *stim* and *sham* group did not differ throughout the experiment, as illustrated in Figures 3AII, BII. To confirm this result, Two-Way ANOVAs with repeated measurements with the between subject factor *group* (2 levels) and the within subject factor *block* (3 levels) were performed. The ANOVAs revealed no significant effects neither for reaction times (*group*: *F*_1_ = 0.19, *P* = 0.67; *time*: *F*_2_ = 0.95, *P* = 0.395; *group* × time: *F*_2_ = 0.167, *P* = 0.80) nor sensitivity *d'* (*group*: *F*_1_ = 0.04, *P* = 0.84; *time*: *F*_2_ = 1.141, *P* = 0.65; *group* × time: *F*_2_ = 2.47, *P* = 0.097).

Sensitivity analysis confirmed that both groups were able to perform the detection task. The absence of significant *group* effects on the behavior confirm that sham stimulation was successful.

#### Electrophysiological data

Endogenous power in the alpha range was enhanced after tACS, but not after sham stimulation (Figure 4AII). However, tACS and sham group did not differ with regard to alpha topography (*r* = 0.97, *P* < 0.001) as illustrated in Figure 5B. The difference between *stim* and *sham* group with regard to the increase in IAF alpha power after stimulation is also visible over time, as illustrated in Figure 4BII. This was confirmed by a Two-Way ANOVA with repeated measurements on the normalized IAF power with the between subject factor *group* (2 levels) and the within subject factor post EEG *time* (10 levels). Significant main effects of *group* (*F*_1_ = 5.84, *P* = 0.025, η^2^ = 0.23) and *time* (*F*_4.71_ = 6.86, *P* < 0.001, η^2^ = 0.26) were observed, but the interaction *group × time* was not significant (*F*_4.71_ = 1.52, *P* = 0.19). No *group* effects were found for the *lower* (*F*_1_ = 0.21, *P* = 0.96) and *upper* frequency band (*F*_1_ = 1.67, *P* = 0.21). Additionally, no difference in the topographies of IAF power between groups could be demonstrated.

Subsequent one-sample *t*-tests against 1 (corresponding to baseline IAF power) revealed that IAF power is enhanced compared to base line after tACS (*t*_10_ = 4.28, *P* = 0.002, *d* = 1.27) but not after sham stimulation (*t*_10_ = 2.13, *P* = 0.06) as illustrated in Figure 4CII. On average, the normalized IAF power after tACS is 148 ± 37%. The effect is not found for the *lower* (*stim*: *t*_10_ = 1.38, *P* = 0.19; *sham*: *t*_10_ = 1.82, *P* = 0.10) and *upper* frequency band (*stim*: *t*_10_ = 1.97, *P* = 0.08; *sham*: *t*_10_ = 0.55, *P* = 0.59).

The significant main effect of *group* demonstrated that *stim* and *sham* groups significantly differed with regard to their IAF power increase. Additionally, compared to baseline, only the group that received tACS had increased IAF power. To get detailed information on how long the IAF power was increased in the *stim* group, we performed one-sample *t*-tests (Bonferroni corrected) against 1 (corresponding to baseline IAF power) for each timepont of the *post* stimulation block. This procedure revealed that the IAF power of the *stim* group did not return to baseline during the whole timecourse of the *post* stimulation block (all *P* < 0.05, *d* > 1.15).

A Two-Way ANOVA with repeated measurements with between subject factor *group* (2 levels) and within subject factor *electrode* (4 levels) revealed a significant main effect of *electrode* (*F*_3_ = 2.98, *P* = 0.038, η^2^ = 0.13). The main effect of *group* (*F*_1_ = 3.14, *P* = 0.08) did not reach significance, but the interaction *groups* × electrode (*F*_3_ = 2.588, *P* = 0.06) showed a trend. The interaction fails to meet the 5% level of significance only by a small margin, indicating that the alpha power increase was indeed locally specific. *Post-hoc t*-tests revealed that IAF power differed significantly only at electrode Pz between groups (*P* < 0.05, *d* = 1.08), differences at the other electrodes were not significant (Fz: *P* = 0.38, FT9: *P* = 0.52, FT10: *P* = 0.38).

Alpha coherence did not differ between groups (Figures 6A lower/B right). This was confirmed by a Two-Way ANOVA with repeated measurements on the normalized alpha coherence with the between subject factor *group* (2 levels) and the within subject factor post EEG *time* (10 levels). Neither significant main effects of *group* (*F*_1_ = 1.35, *P* = 0.26) and *time* (*F*_3.95_ = 1.04, *P* = 0.39) nor a significant interaction *group* × time (*F*_3.95_ = 0.72, *P* = 0.58) was observed.

In order to further elaborate on the coherence effect in the tACS stimulation groups, we correlated the *pre* stimulation coherence for all subjects of both eyes open and eyes closed tACS stimulation groups with their average *post* stimulation change in coherence (Figure 6C). The correlation was *r* = −0.33, which means that the lower *pre* stimulation coherence the higher the increase in the *post* stimulation period. However, the correlation was not significant (*P* = 0.145).

For a demonstration that the physiological after-effect of tACS differed for the eyes closed experiment compared to the eyes open experiment, additional Three-Way ANOVAs were conducted with between subject factors *condition* (2 levels; eyes closed/open) and *group* (2 levels; stim/sham) and the within subject factor *time* (10 levels). The interaction *condition* × group was significant for both ANOVAs (*power*: *F*_1_ = 5.104, *P* = 0.012, η^2^ = 0.16; *coherence*: *F*_1_ = 6.841, *P* = 0.013, η^2^ = 0.16).

## Discussion

We have reported novel findings regarding the duration of frequency-specific after-effects of tACS stimulation in endogenous EEG power. In addition, evidence is provided that the endogenous oscillatory power of the entrained frequency has a crucial impact on the efficacy of tACS.

Endogenous IAF power before (baseline) and 30 min after tACS was compared. Participants had their eyes either closed (high endogenous IAF power) or open (low endogenous IAF power). In the eyes closed experiment, no effects on oscillatory power were observed, i.e., IAF power did not significantly differ from pre-stimulation levels neither in the sham nor the stim group. Contrary to the results in the eyes closed experiment, an effect on the oscillatory power, limited to the alpha range, was found in the eyes open experiment. The power increase from the pre- to the post stimulation period was significant in the stimulated group, but not in the sham group. This effect lasted for the complete duration of the 30 min post-stimulation recording period.

Via the application of tACS, we intended to modulate alpha oscillations; however, under conditions of high endogenous alpha power, tACS fails to influence alpha power. One possible explanation to account for this finding is that with eyes closed, endogenous alpha power has reached a maximum (Nunez et al., 2001) and cannot be further enhanced by tACS, due to ceiling effects. This hypothesis cannot be rejected with respect to our results. A second, more mechanistic, explanation would suggest that the low oscillating currents introduced to the brain by tACS are not strong enough to modulate high endogenous oscillations, but only low endogenous oscillations. From a recent simulation study, we know that 1 mA of tDCS/tACS results in an electric field of 417 μV/mm in occipital areas (Neuling et al., 2012b). Intracranial recordings taken from the visual cortex of wake behaving monkeys performing a task revealed alpha powers in the range of 400 μV/mm (Bollimunta et al., 2008), which is almost identical to the maximum voltage gradient resulting from tACS in occipital cortex. Since monkeys had their eyes open during the experiment, we expect that these values represent the case of low endogenous alpha power. With closed eyes, the power of endogenous alpha oscillations are increased by a factor of 2.27 ± 0.93 compared to the power with open eyes (Könönen and Partanen, 1993). This suggests stimulation intensities more than twice as high as used by us are required to enhance alpha powers in the eyes closed condition. Although this mechanistic explanation is intriguing, further studies with variable stimulation power are required to rule out the ceiling-effect explanation. Our results demonstrate that endogenous oscillations exhibiting lower power before tACS are more prone to the effects of tACS and are enhanced over a long period of time. It might be speculated that the enhancement effect would last even longer than 30 min.

It is interesting to compare electrical stimulation with repetitive sensory stimulation. It has been demonstrated repeatedly that flickering light is able to entrain brain oscillations in form of so-called visually steady-state evoked potentials (SSEP; Regan, 1989). Systematic variation of the driving frequency has revealed a resonance peak in the SSEP at subject's IAF (Herrmann, 2001). Intriguingly, this resonance effect occurs particularly in subjects with high alpha power (Pigeau and Frame, 1992). In contrast to their study, we were only able to achieve entrainment with eyes open, i.e., at low alpha power. At first glance, this seems like a contradiction; however, high and low alpha power between subjects due to eye opening is not comparable to inter-individual variations of alpha power. Even subjects with low alpha power will show variations due to eye opening. Nevertheless, the study by Pigeau and Frame (1992) demonstrates that visual stimulation has a stronger effect on visual cortex than our electrical stimulation, since even high alpha subjects could be entrained.

It may be argued that the observed effects of increased IAF power in the eyes open experiment do not result from tACS, but instead result from sensory deprivation and the monotonous task. This possibility can be ruled out by our data, because the IAF power of the *sham* group was not significantly enhanced in the post stimulation block. It is also unlikely that tACS increases tiredness and had resulted in increased alpha power, because ratings on the questionnaire items *tiredness* and *trouble concentrating* did not differ between groups, neither in the eyes open, nor in the eyes closed experiment.

A further concern about tACS effects in the visual cortex is whether they are of cortical or retinal origin (e.g., Schwiedrzik, 2009). We used the same electrode montage as Kanai et al. (2008). The authors reported phosphenes thresholds in a dark room at 500 μA stimulation intensity at alpha frequency; however, Kanai et al. (2008) used a smaller electrode over the occipital cortex compared to the current study (3 × 4 cm vs. 5 × 7 cm) leading to a higher current density under the stimulation electrode. Their current density was 42 μA/cm^2^, which exceeds our highest density of 29 μA/cm^2^ that was reached at a stimulation intensity of 1000 μA. This means, with our stimulation intensity, we were below the phosphene threshold, which was additionally confirmed by our questionnaire results. Furthermore, Kar and Krekelberg (2012) demonstrated that even with occipital electrode montage, perceived phosphenes are of retinal origin. Nevertheless, as no phosphenes were reported in the current study, we assume that the long lasting after-effects are due to direct cortical modulation.

The current results further support existing evidence for successful entrainment of oscillatory brain activity (Thut et al., 2011a). We applied tACS at a frequency that was predominant in the spontaneous brain activity of the individual subjects and our results demonstrate that we successfully enhanced the spontaneous activity in a frequency-dependent manner. Furthermore, topographies demonstrate that tACS effects on alpha power are local and enhance alpha oscillations that could be observed before stimulation (cf. Figure 5). One of the main questions is, how tACS of the intensity used in our study is able to modulate the neuronal network activity at all. The electrode configuration used in this study targeted the occipito-parietal alpha rhythm (Nunez et al., 2001). A modeling approach by Neuling et al. (2012a) demonstrated that this electrode layout is well suited to affect the occipital cortex (cf. Figure 1). The authors report that TES with an intensity of 1 mA, similar to the intensities used in the current study, results in currents of 420 μV/mm in gray matter, which is of sufficient strength to modulate neuronal network activity in the ferret visual cortex (Fröhlich and McCormick, 2010).

**Figure 7:**
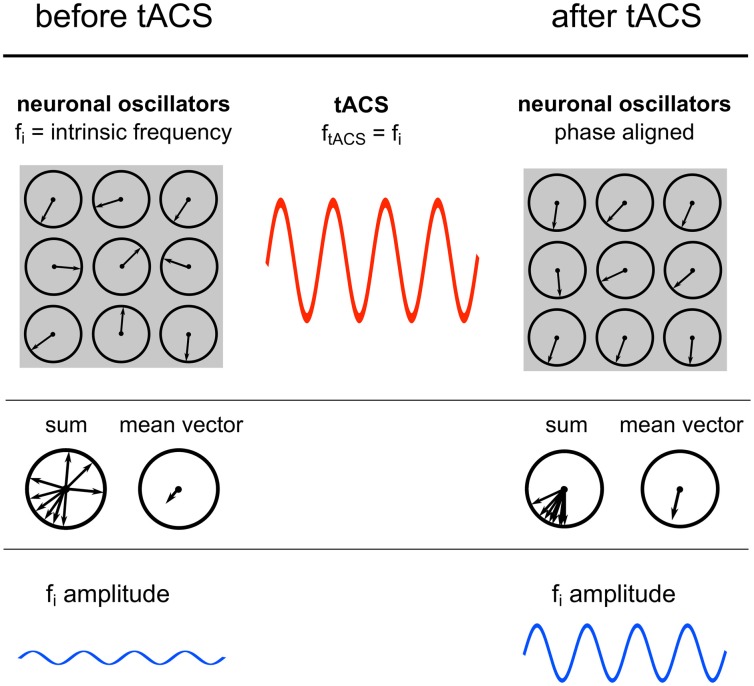
**Power increase by synchronization.** Illustration of the proposed underlying neuronal mechanism of the power effect. **Upper row:** Single neuronal oscillators, depicted as vectors on a unit circle, with a specific intrinsic frequency synchronize to an external sinusoidal force with the same frequency. **Middle row:** The mean resultant vector of the single oscillating elements represents the amplitude of the oscillation. Due to phase alignment of each single oscillator to the external oscillation, the length of this mean vector increases and likewise the amplitude. **Lower row:** Schematic illustration of the amplitude recorded via EEG.

Sinusoidal electric fields generate periodic states of depolarization and hyperpolarization relative to the resting membrane potential of individual neurons, thereby generating periods of higher and lower firing probability of the neuron and consequently guiding the entire neuronal population (Fröhlich and McCormick, 2010). The authors demonstrated that although this effect is small on individual neurons it has a strong effect on network dynamics. During continuous stimulation with an oscillating current, spontaneous activity of an increasing number of individual neurons starts to synchronize^1^ with the external oscillation, which in turn results in a power increase of the whole network (Figure 7). These findings are congruent with the current study, where elevated power levels have been reported.

A population of simple oscillators with similar intrinsic frequencies can be entrained by a single external oscillator (Winfree, 1980). This also applies for neuronal oscillators. An ideal condition for entrainment is a frequency match between the periodic external force and the intrinsic oscillation (Hutcheon and Yarom, 2000; Pikovsky et al., 2003). This means that neurons oscillating in the range of the external stimulation frequency will be entrained but neurons with intrinsic frequencies outside the stimulation frequency will not be affected (Fröhlich and McCormick, 2010). This is confirmed by the result that only IAF power is increased, whereas, surrounding frequency bands remain unaffected.

Unfortunately it is not yet possible to record EEG online during tACS due to the artefact induced by the electric stimulation current. This would yield important insights into the mechanism of action of tACS and its immediate effect on the EEG and the dynamics of brain functions (Miniussi et al., 2012).

Sustained entrainment after the end of oscillatory TES in the alpha range has been reported by Zaehle et al. (2010) and Neuling et al. (2012a). However, these studies only analyzed 3 min directly after the stimulation. The current study demonstrates that these after-effects can outlast the stimulation for at least 30 min. Compared to Zaehle et al. (2010), who also targeted the occipital cortex with tACS at IAF, the current study found a stronger increase of individual IAF power (mean increase: 14% vs. 48%). This difference could be explained by two important parameters: Zaehle et al. (2010) stimulated for 10 min while we applied 20 min of tACS. Furthermore, we used a medial electrode configuration compared to the bilateral configuration used by Zaehle et al. (2010). A medial configuration might be beneficial to synchronize both hemispheres at 0° which might lead to the more pronounced power effect, while bilateral stimulation will lead to a phase difference of 180° between hemispheres.

We found a significant difference in the increase of alpha coherence between groups in the eyes closed condition. This difference was not found in the eyes open condition. One explanation for this effect might be a ceiling effect: if the coherence is already high before stimulation, it does not increase further. We found a negative correlation between the coherence before stimulation and the relative coherence after stimulation. This result suggests that pre stimulation coherence modulates the effect, but the correlation did not reach statistical significance. Although our hypothesis that the coherence before stimulation affects the increase after stimulation cannot be confirmed by our results, the negative correlation encourages further experiments to reveal the underlying mechanism of the coherence effect. Interestingly, results of another recent study of ours are in line with the data of the eyes closed condition (Strüber et al., under revision). We were only able to influence to coherence, but not the amplitude of EEG oscillations.

So far, after-effects of tACS up to an hour post-stimulation have only been reported regarding excitability, measured via motor evoked potentials (MEP) evoked with transcranial magnetic stimulation (Moliadze et al., 2010; Wach et al., 2012). However, EEG was not recorded in these studies, thus no statements can be given regarding effects on EEG power. After-effects lasting up to 90 min have been reported after tDCS (Nitsche and Paulus, 2001). After 13 min of stimulation with 1 mA, cerebral excitability enhancement up to 90 min has been detected via TMS evoked MEPs. Even though EEG was not recorded, these findings are in line with the duration of our effect.

A possible neurophysiological mechanism accounting for the long term after-effects in our study is based on synaptic plasticity (Zaehle et al., 2010). By means of spike-timing-dependent-plasticity (Markram et al., 1997), synapses can be strengthened, a mechanism known as long term potentiation or weakened, referred to as long term depression. During stimulation, synapses in neuronal circuits with an intrinsic frequency (Hutcheon and Yarom, 2000) of the external oscillation (tACS) are strengthened and are thought to persist after stimulation. This assumption was supported by network simulations (Zaehle et al., 2010), which resulted in enhanced synaptic weights of those synapses that were incorporated into neural loops whose frequency was close to that of tACS.

The duration of the after-effects presented here is a prerequisite for clinical applications of tACS for diseases correlated with altered oscillatory brain activity. For example, in schizophrenia, a dysregulation of oscillatory gamma activity may correlate with distinct schizophrenic symptoms (Lee et al., 2003). In Parkinson's disease, abnormal beta activity can lead to motor slowing (Hammond et al., 2007). Furthermore, evoked gamma-band activity is altered in children with attention deficit hyperactivity disorder (Lenz et al., 2010).

One can conclude that tACS has the potential to regulate brain dysfunctions that are related to EEG frequencies. Long lasting changes of endogenous brain oscillations could be used to balance modified brain oscillations. Furthermore, the knowledge that endogenous power has effects on tACS efficacy can be employed to improve attempts of task-related modulations of brain rhythms to modify behavior and improve learning.

### Conflict of interest statement

The authors declare that the research was conducted in the absence of any commercial or financial relationships that could be construed as a potential conflict of interest.

## Footnotes

^1^Note that an oscillator can be phase-locked and/or frequency-locked to an external driving force. Frequency-locking can occur without phase-locking, but phase-locking implies also frequency-locking (Izhikevich, 2007, p. 460); however, our data cannot differentiate between these two states of synchronization.
